# Role of Cystic Lymph Nodes in Incidental Gallbladder Cancer: Implications for Routine Excision and the Sentinel Lymph Node Concept

**DOI:** 10.7759/cureus.99104

**Published:** 2025-12-13

**Authors:** Andreas Efstathiou, Spiros Delis, Ajay Sharma, Kanwhal Jeet Singh, Vinay Kumar Kapoor

**Affiliations:** 1 General Surgery, Konstantopoulio General Hospital, Athens, GRC; 2 Surgery, University of Miami, Miami, USA; 3 Surgical Gastroenterology, Mahatma Gandhi Medical College and Hospital, Jaipur, Jaipur, IND; 4 Gastrointestinal Surgery, Healing Touch Hospital, Jalandhar, Jalandhar, IND; 5 Surgical Gastroenterology, Sanjay Gandhi Postgraduate Institute of Medical Sciences, Lucknow, IND

**Keywords:** cystic lymph node, gallbladder neoplasm, incidental gallbladder cancer, laparoscopic cholecystectomy, lymphadenectomy, lymph node dissection, sentinel lymph node, simple cholecystectomy

## Abstract

Gallbladder cancer (GBC) is a rare but aggressive malignancy, often diagnosed incidentally after or even during routine cholecystectomy for presumed benign conditions, the commonest being gallstone disease (GSD) - this is incidental GBC (iGBC). The involvement of cystic lymph nodes (CLNs), which are located near the cystic artery and the cystic duct, has been an area of clinical interest in predicting the prognosis, staging, and therapeutic decision-making of GBC. This review explores the significance of CLN status in iGBC, evaluates the potential benefits of routine CLN excision during simple cholecystectomy, and explores the applicability of the sentinel lymph node (SLN) concept in the management of iGBC.

## Introduction and background

Gallbladder cancer (GBC) carries a poor prognosis and is often diagnosed at an advanced stage [1]. Its global incidence is low (approximately 1-2 per 100,000 in the West) but varies geographically, with higher rates in regions such as Chile, North India, and among certain ethnic groups [2]. Even with aggressive therapy, overall five-year survival remains in the single digits to low teens (5-12%). An important subset of patients is those with incidental gallbladder cancer (iGBC) - defined as cancers unexpectedly discovered on pathologic examination of a cholecystectomy specimen removed for presumed benign conditions. In Western countries, the majority of GBC cases are now identified incidentally after routine gallbladder surgery. For example, up to ~3% of cholecystectomies may harbor an unanticipated GBC, and in one series, 72% of new GBC diagnoses had a recent cholecystectomy for another indication. Compared to overt GBC, incidentally detected tumors are often lower grade and stage, offering a window of opportunity for curative intervention if managed appropriately [3].

Management of iGBC typically involves a two-stage approach. The initial cholecystectomy (often laparoscopic) is followed by a prompt re-operation (re-resection) for patients with tumors beyond the mucosa (stage T1b or higher). The goal of re-resection is to obtain clear margins and remove regional lymph nodes for staging and potential cure. An extended cholecystectomy is the recommended standard for T2 or greater lesions: this includes a partial hepatic resection (segments IVB/V or a wedge of the gallbladder bed) and an extensive lymphadenectomy of the hepatoduodenal ligament [4]. Notably, clinical guidelines and surgical oncologists emphasize that the lymphadenectomy in GBC should encompass the cystic duct lymph node (Calot’s node) along with nodes along the common bile duct, hepatic artery, portal vein, and even posterior superior pancreaticoduodenal nodes [5]. This is because accurate nodal staging is crucial - retrieval of at least six lymph nodes is often recommended for proper staging in GBC [6]. Patients with node-positive disease have significantly worse outcomes than node-negative patients, and the number of positive nodes further stratifies prognosis (e.g., ≥4 positive nodes portend especially poor survival) [7]. Thus, complete regional lymph node dissection (RLND) at reoperation is considered standard to improve staging accuracy and potentially survival.

However, the necessity and extent of lymph node dissection in iGBC have been areas of debate. Lymphadenectomy adds complexity and potential morbidity to an already major reoperation. Particularly in elderly or comorbid patients, surgeons may hesitate to perform a full RLND due to higher operative risks [8]. In fact, there are instances where recommended re-resections are not undertaken in older patients - in one study from a Japanese single-institution series with a median age of 80 years, six iGBC patients had pT2-pT3 disease, yet only one underwent additional resection, with most elderly patients deferring further surgery [9]. This underscores a clinical dilemma: while oncologic principles favor extended resection, a tailored approach might be preferable for select patients. Here is where the concepts of the cystic lymph node (CLN)’s role and the “sentinel lymph node (SLN)” strategy enter the discussion.

Objectives

This narrative review aims to critically assess the role of the CLN in the context of iGBC. We focus on whether routine excision and analysis of the CLN can aid in staging and guiding management of iGBC, and we evaluate the evidence supporting the SLN concept in GBC. In essence, can the status of the cystic node reliably indicate the status of other regional nodes, thereby influencing decisions about the extent of surgery? We synthesize current literature (especially from the past 5-10 years) on anatomical lymphatic pathways, outcomes related to CLN involvement, the feasibility of SLN mapping in GBC, and the potential clinical implications of incorporating these concepts into practice.

This article is based on the presentations and discussions held at the 3rd Jaipur Surgical Festival (JSF) - International Study Group of Gall Bladder Cancer (ISG-GBC), organized at Mahatma Gandhi Medical College and Hospital (MGMCH), Jaipur, Rajasthan, India, from 1-3 December 2023.

## Review

Methodology

A narrative literature review was performed to gather relevant evidence on CLNs and the sentinel node concept in GBC. We searched databases including PubMed and Google Scholar for English-language articles up to 2025, using keywords such as “incidental gallbladder cancer,” “cystic duct lymph node,” “sentinel lymph node biopsy gallbladder,” and “gallbladder cancer lymphadenectomy.” Priority was given to recent (last 10 years) peer-reviewed studies, clinical series (retrospective and prospective), systematic reviews, and published guidelines that specifically addressed lymph node involvement or surgical management in GBC. Older foundational studies were included if they provided anatomical or historical context. Given the rarity of GBC, high-level evidence is limited; thus, this review synthesizes data from multi-institutional retrospectives, population-based analyses, and expert surgical guidelines to provide a comprehensive overview.

Epidemiology and clinical context of incidental GBC

GBC has an unusual epidemiology marked by geographical and demographic disparities. Certain populations (e.g., Chileans, Native Americans, Northern Indians) experience a higher incidence, likely due to genetic and environmental factors, while in most Western countries, GBC is rare [1,2]. Regardless of region, the disease is often aggressive and lethal when advanced, but outcomes improve dramatically if it is caught early and managed surgically. The widespread adoption of laparoscopic cholecystectomy over recent decades has inadvertently led to more frequent detection of early-stage GBC. Patients undergo gallbladder removal for common benign conditions (like gallstones or cholecystitis), and a surprise cancer is found in the pathology in anywhere from 0.5% to 3% of these cases. Indeed, incidental GBC now accounts for the majority of GBC presentations in low-incidence countries [3].

The “incidental” nature of the diagnosis presents unique challenges. The patient’s first operation is not planned as an oncologic procedure, so the margins and lymph nodes are typically not adequately addressed at that time. Once iGBC is diagnosed postoperatively, the standard of care is to stage the disease and consider a completion surgery (re-resection) for curative intent [4]. Re-resection is generally indicated for all tumors invading beyond the lamina propria (i.e., T1b or higher) in patients who are fit for surgery. This completion surgery usually entails an extended cholecystectomy, which includes hepatic resection of the gallbladder bed and an RLND. The rationale is to remove any residual microscopic disease left in situ and to harvest lymph nodes for staging and improving locoregional control [10]. Studies have shown that residual disease (either in the liver or nodes) is present in a significant proportion of iGBC cases at reoperation due to occult microscopic spread left behind after a non-oncologic index cholecystectomy - including unrecognized hepatic-side invasion of the gallbladder bed, positive/close cystic duct margins, and undissected regional lymphatics - often compounded by adverse biology (≥T2 stage, lymphovascular/perineural invasion) and, in some cases, bile spillage or gallbladder perforation during the initial operation. Clearing this disease can improve survival chances [11].

Crucially, the status of lymph nodes is one of the strongest prognostic determinants in GBC. Patients with node-negative re-resections have far better survival than those with nodal metastases. For example, Vega et al. reported a three-year overall survival of around 81% in node-negative iGBC patients versus 37% if one to three nodes were positive and only ~18% if ≥4 nodes were positive. This reflects the known staging classification, where N2 disease (extensive nodal spread) is considered stage IV with a very poor prognosis. Therefore, thorough lymphadenectomy and accurate nodal staging are critical [12]. Current surgical guidelines recommend dissecting the lymph nodes of the hepatoduodenal ligament, including the cystic duct node, common bile duct nodes, nodes around the hepatic artery and portal vein, and retropancreatic (posterior superior pancreaticoduodenal) nodes as necessary. In practice, achieving a harvest of at least six lymph nodes is advised for proper staging, and this lymph node count is also used as a quality indicator for GBC surgery [13,14].

While extended resection is the gold standard, it comes with a cost. The additional liver resection and lymph node dissection significantly increase operative time and complexity, and patients face higher risks of complications such as bile leak, liver failure, or abscess. This is especially relevant for older patients: iGBC is often discovered in patients in their 60s or 70s (since gallstone disease incidence increases with age), and these individuals may have comorbidities that make a second major surgery high risk [15]. There is evidence that surgeons sometimes forego re-resection in very elderly patients or those with limited life expectancy. A Japanese series noted that in a cohort of iGBC patients (median age 80), many did not undergo the recommended completion surgery due to advanced age or limited resources. This scenario has prompted interest in more selective surgical strategies - identifying which patients truly benefit from re-resection and how to minimize surgery while still effectively treating the cancer. If we could accurately determine by sampling that certain patients (e.g., those without lymphatic spread) are unlikely to have residual disease, perhaps some re-resections could be avoided or reduced in scope [9].

In this context, the concept of the CLN as a sentinel node is highly relevant. The CLN is often the first node encountered in a standard cholecystectomy, and if it could be sampled or analyzed at the time of initial surgery (or via minimally invasive means soon after surgery and histologically proven malignancy), its status might inform the need for further intervention and guide towards operative surgery [12,16]. Before analyzing that concept, it is important to understand the anatomical basis of gallbladder lymphatic drainage and why the cystic node has been considered a key player.

Anatomical basis and lymphatic drainage of the gallbladder

The gallbladder has a rich lymphatic network in its subserosal layer that drains into regional lymph nodes. The CLN - also known as the node of Lund - is a small lymph node typically situated in Calot’s triangle (the triangle formed by the cystic duct, common hepatic duct, and the inferior edge of the liver). It lies adjacent to the cystic artery and cystic duct, often on the posterolateral aspect of the cystic duct [17]. Because the CLN lies in the adipose tissue between the cystic duct and cystic artery, its removal or preservation reflects how surgeons dissect the hepatobiliary triangle. Wysocki et al. demonstrated wide variation in CLN excision rates among surgeons (0-50%), suggesting that the node occupies a consistent anatomic position but is intercepted only when the dissection plane passes medially [18]. One retrospective study reported that the cystic node was visually identified in approximately 78% of laparoscopic cholecystectomies, and in nearly half of cases, the dissection could be kept lateral to the node entirely (thus protecting the bile duct). This highlights that the CLN is both conspicuous and potentially useful during surgery [19].

From an oncologic standpoint, the CLN is classically thought to be the first echelon lymph node draining the gallbladder [16]. Submucosal and subserosal lymphatic channels from the gallbladder wall converge toward the neck of the gallbladder and Calot’s triangle. Lymph then flows into the cystic node along the path of the cystic artery. After the cystic node, lymph from the gallbladder can travel along two principal routes in the hepatoduodenal ligament:

Right-Sided (Posterior) Route

This is considered the dominant pathway for gallbladder lymphatic drainage. Lymph passes from the CLN to nodes along the common bile duct and portal vein (often referred to as pericholedochal or hilar nodes), and then to the retroportal and pancreaticoduodenal nodes, and further to the interaortocaval lymph nodes (around the aorta/caval bifurcation). In other words, it goes downward and posteriorly toward the pancreatic head and then centrally.

Left-Sided (Anterior) Route

An alternate pathway carries lymph from the region of the cystic node toward the nodes around the hepatic artery and then to the celiac axis and para-aortic lymph nodes. This route runs more along the anterior hepatoduodenal ligament and upward to the celiac region [17,20].

Because of these multiple pathways, gallbladder carcinoma can spread to a variety of nodal stations. The regional lymph nodes for GBC (as defined by the American Joint Committee on Cancer (AJCC)) include nodes in the cystic duct area, the hepatoduodenal ligament (porta hepatis), along the common bile duct, hepatic artery, and behind the pancreas (posterior superior pancreaticoduodenal nodes). More distant nodes like celiac, superior mesenteric, and para-aortic nodes are considered distant metastases (M1) in some staging systems, or “N2” disease in older staging schemes, reflecting advanced spread [21].

A key implication of this anatomy is that the CLN is not invariably involved even when other regional nodes are positive. In other words, while the CLN is a primary waypoint, it is not a perfect gatekeeper. Metastatic cancer cells from the gallbladder can sometimes bypass the CLN and travel via alternate channels to reach second-tier or third-tier nodes. Autopsy and surgical studies of lymphatic drainage have confirmed that in some cases of GBC, lymphatic spread can occur directly to the hilar (porta hepatis) nodes or to nodes along the hepatic artery without metastasis in the cystic node. In one anatomical study, it was observed that subserosal lymphatics of the gallbladder can drain directly into the liver’s subcapsular lymphatics, providing another route for tumor spread into hilar nodes or the liver parenchyma [12,20]. In practice, this means a patient could have a negative CLN but still harbor metastases in other regional lymph nodes - a phenomenon known as skip metastasis. Conversely, a positive CLN will usually (but not always) indicate further nodal spread beyond it, simply because if tumor cells reached the first node, they may have continued onward [16].

Understanding these lymphatic pathways sets the stage for evaluating the SLN concept in GBC. In cancers like melanoma and breast cancer, the SLN biopsy concept relies on a consistent, orderly lymph drainage where the first node can serve as an indicator for the rest of the basin [22,23]. GBC’s lymphatic spread is more complex, but the question is whether the cystic node can serve as a reliable sentinel under certain conditions. Before addressing that, we will examine what is known about the prognostic significance of the CLN itself in GBC.

Prognostic role of CLNs in GBC

Lymph node metastasis is one of the most significant prognostic factors in GBC. This holds true for incidental cases as well as for those detected upfront. Any involvement of lymph nodes generally upstages the disease to stage III or IV (depending on the number of nodes) and is associated with a marked decrease in survival. As noted, analysis of large case series shows stark differences in outcomes: patients with node-negative (N0) GBC have a much higher chance of long-term survival than those with node-positive disease. For example, in a combined dataset of incidental GBC patients who underwent curative re-resection, three-year survival was on the order of 69-81% for N0 patients, but only ~30-40% in those with N1 (one to three positive nodes) and as low as 15-20% for N2 (≥4 positive nodes). These figures illustrate the steep prognostic gradient associated with nodal status [7,12].

Where does the CLN fit into this picture? Essentially, the CLN is one of the N1 nodes (first-tier nodes) and often the first to be involved if metastasis occurs. If the final pathology of a resected specimen shows the cystic node is positive, the patient is, by definition, node-positive (at least N1). If it is the only positive node and others are negative, one might consider that a limited nodal disease scenario (which might have a better prognosis than multiple positive nodes). Historically, some data suggested that metastasis confined to the cystic node (with no spread beyond) might carry an intermediate prognosis between N0 and widespread N+ disease. However, truly isolated cystic node metastasis is relatively uncommon by the time of completion of surgery because if the tumor had the capability to spread, it often has reached other nodes too [20].

Several studies have specifically looked at the predictive value of the cystic node. One notable multi-center study addressing this is by Vega et al. (2018), which focused on incidental GBC and lymph node status. In their analysis of 187 iGBC patients from centers in the United States and Chile, the cystic duct node (referred to as Calot’s node, or pN12c in some notations) was retrieved in 40% of cases (indicating it is not always submitted or found in pathology). They found that if the cystic node was positive, there was a very high likelihood that the hepatoduodenal ligament nodes (the next tier, often called the N1 nodes in older classification) were also positive - in fact, a positive CLN predicted hilar (N1) node metastasis with an odds ratio of ~22. This underscores that a positive CLN is a strong marker of further regional spread. On the other hand, Vega et al. showed that a negative cystic node did not guarantee the absence of other nodal metastases in all cases. They documented skip metastases: patients who had no tumor in the CLN but did have positive nodes in downstream locations (e.g., cystic node negative, but a common hepatic artery node or para-aortic node positive). In their series, about 12.8% of patients demonstrated such skip nodal metastasis despite a negative CLN. Therefore, the cystic node status alone was insufficient to rule out further nodal involvement.

Importantly, Vega and colleagues evaluated outcomes based on cystic node status. They observed that if the cystic node was positive, the disease was generally more advanced and the prognosis worse (as expected). However, they made an intriguing observation: among patients who underwent a thorough re-resection and lymphadenectomy, those who had only the cystic node positive but all other nodes removed were negative had a similar disease-specific survival to those who were entirely node-negative. In other words, if a positive cystic node was the only site of nodal metastasis and the extended operation cleared everything else, the outcome could be as favorable as if the patient had no nodal metastasis at all. This finding suggests that the prognostic impact of a positive CLN can be mitigated if it is an isolated metastasis and it is removed. Unfortunately, in many cases, a positive CLN is accompanied by additional positive nodes, which drives prognosis down [12,16].

The prognostic role of CLN is also intertwined with tumor location in the gallbladder. There has been increasing evidence that tumor location (anatomical site on the gallbladder wall) influences patterns of spread and outcomes. Tumors on the hepatic side of the gallbladder (i.e., those involving the portion of the gallbladder attached to the liver) have a higher propensity for direct liver invasion and also for lymphatic spread into the liver and hilar nodes. Tumors on the peritoneal side (the free side facing the abdominal cavity) tend to present a bit earlier (as they can cause symptoms or be noticed due to serosal involvement) and have a lower rate of LN metastasis. A landmark international study by Shindoh et al. (2015) and others has shown that for T2 GBCs, hepatic-side tumors had significantly worse survival than peritoneal-side tumors. One major reason is that hepatic-side tumors more frequently have positive LNs and possibly microscopic liver invasion. This is relevant to the CLN because the cystic node drains the region of the gallbladder neck and cystic duct. A fundus or body tumor (peritoneal side) might have to travel a bit to reach the cystic node, whereas a tumor growing into the liver might spread through intrahepatic lymphatics to hilar nodes, sometimes bypassing the cystic node [24].

Recent data from Yasukawa et al. (2021) shed light on this. They stratified GBC patients by tumor location and examined the relationship between the cystic duct node and other nodal metastases. In their study, no patient with a T2 tumor on the peritoneal side (T2p) had downstream node metastasis if the cystic node was negative. In contrast, for T2 tumors on the hepatic side (T2h), they documented several cases of skipped metastases (cystic node negative but other LNs positive). Specifically, three out of 20 T2 hepatic-side tumors had this skip pattern despite a negative CLN. For T3 tumors, skip metastases were even more frequent, especially on the hepatic side (11 of 20 T3h cases). Given this strong stage- and side-dependence, the reliability of CLN as a sentinel varies across subgroups (Table 1). These findings confirm that the reliability of the cystic node as a predictor of overall nodal status is highly contingent on tumor location and depth [16].

**Table 1:** Tumor location versus reliability of CLN sentinel concept. "Downstream LN+” refers to hepatoduodenal ligament/hilar/CHA/retropancreatic nodes depending on tier. CLN: cystic lymph node; SLN: sentinel lymph node; LN: lymph node; LND: lymph node dissection; p: peritoneal side; h: hepatic side; NR: not reported; CHA: common hepatic artery

Stage subgroup	CLN– → downstream LN+?	Skip rate	Reliability of CLN as SLN	Recommended surgery (your conclusion aligned to evidence)
T1b (any side)	Possible but uncommon; evidence limited	NR/low	Not established	Radical cholecystectomy + regional LND for staging; don’t rely on CLN alone.
T2p	No (0 cases in Yasukawa et al.)	0%	High/SLN concept valid	If CLN is negative and margins are clear, consider limited LND in carefully selected patients; still controversial.
T2h	Yes	15% (3/20)	Moderate-low	Standard radical re-resection + D2 LND regardless of CLN.
T3p	Yes	9.1% (2/22)	Low	Radical resection + D2 LND. Negative CLN is not reassuring enough.
T3h	Yes, frequently	55% (11/20)	Very low/unsafe to use as SLN	Aggressive resection + D2 LND + systemic therapy consideration.

In summary, a positive CLN in GBC almost always signifies advanced disease with further nodal spread (and worse prognosis), while a negative CLN is a favorable sign but does not guarantee an absence of metastasis in others, particularly for more advanced or unfavorable tumor locations. The prognostic utility of the CLN, therefore, is significant but not absolute. This has led to exploration of whether the cystic node could be used as an SLN - meaning, if it’s negative, could we safely assume the rest of the regional nodes are negative (and thus perhaps avoid a full lymphadenectomy in some cases)? We delve into that next.

SLN concept in GBC and current evidence

The SLN concept refers to the idea that the first draining lymph node of a primary tumor can serve as a representative for the status of the entire lymphatic basin. SLN biopsy is an established practice in cancers like breast cancer and melanoma, where a negative SLN spares the patient an extensive lymph node dissection, and a positive SLN directs further nodal surgery or therapy [22,23]. In gastrointestinal cancers, the role of SLN mapping is less straightforward, but it has been investigated in gastric cancer and others with some success [25]. GBC is an attractive but challenging scenario for SLN mapping due to the complex lymph drainage and the need for rapid decisions (since most cases are diagnosed post-hoc).

Historically, the cystic node has been presumed to be the “sentinel” node for the gallbladder by virtue of its location. The question is: if the cystic node is negative, can we omit a full lymphadenectomy (or even avoid reoperation) in iGBC? Conversely, if the cystic node is positive, does that automatically necessitate maximal surgery? Several key studies have tried to answer parts of this question:

Vega et al. (2018)

As discussed, among 187 patients undergoing extended re-resection for iGBC, retrieval of the cystic duct (CLN) node was achieved in ~39%. They observed that a positive cystic duct node (pN12c+) was significantly associated with positive hilar (D1) nodes (odds ratio (OR) 5.2, p = 0.012) but did not reliably predict more distant (D2) nodal involvement. Notably, 12.8% of patients with negative CLN had positive D2 nodes (skip metastasis). Although pN12c+ predicted worse survival overall (hazard ratio (HR) 2.09), patients with isolated pN12c+ and no residual cancer had disease-specific survival comparable to pN0 patients (70% vs. 60%, p = 0.337). Based on these results, the authors concluded that CLN status alone is insufficient to guide the extent of lymphadenectomy, and recommend a formal extended (D2) lymph node dissection (OER) for all patients undergoing re-resection - regardless of CLN status - to ensure accurate staging and optimize oncologic outcomes [12].

Yasukawa et al. (2021)

This Japanese study explicitly tested the SLN concept in GBC. They defined the cystic duct node as the putative SLN and analyzed 80 patients with T2-T3 GBC. The results were strikingly location-dependent. In T2 peritoneal-side tumors (T2p), the sentinel concept held perfectly: if the cystic node was negative, all other regional nodes were negative; if the cystic node was positive, then downstream nodes were invariably positive. In this subgroup, the sensitivity and specificity of the cystic node status for predicting other nodal metastases were 100%. This suggests that for T2 tumors confined to the free (peritoneal) side of the gallbladder, a negative cystic node could potentially assure node-negative status, and extensive node dissection might be safely omitted. In contrast, for T2 hepatic-side tumors (T2h), the sentinel node concept did not fully hold - they had cases of skip metastases (cystic node negative, others positive) and calculated the sensitivity/specificity around 75%. For T3 tumors, the sentinel reliability was even lower on the hepatic side (sensitivity ~80%, specificity ~27%), meaning frequent misclassification, driven mainly by a very low specificity and also some false negatives, making reliance on the cystic node alone unsafe in T3h tumors. Yasukawa et al. concluded that “the concept of SLN can be applicable to T2p GBC” (T2 tumors on the peritoneal side), but not to T2 tumors on the hepatic side or more advanced tumors. They suggested that in carefully selected T2p cases, perhaps one could omit a full lymphadenectomy if the cystic node is negative, thereby sparing the patient an extensive dissection. This is a bold suggestion and would need validation, but it opens the door to a personalized surgical approach based on tumor location and SLN status [16].

Other Studies

Earlier efforts to evaluate SLN mapping in GBC have been limited due to the rarity of early-detected cases. Preclinical work has demonstrated that SLN detection in GBC is technically feasible: in a swine model, Mihara et al. successfully identified SLNs using a dual-tracer approach combining indocyanine green (ICG) and superparamagnetic iron oxide. However, these findings remain experimental and have not yet been translated into consistent clinical practice [26]. More recently, novel techniques are being tried. A 2024 case report by Brañes et al. described the use of ICG fluorescence to identify SLNs during a laparoscopic extended cholecystectomy in an elderly patient. In that case, 1 mL of ICG was injected into the gallbladder bed area (avoiding direct injection into the tumor), and a near-infrared laparoscopic camera was used. The lymphatics draining the gallbladder lit up, and a fluorescent lymph node was seen posterolateral to the bile duct (corresponding to a cystic/hepatoduodenal ligament node, station 12b). The surgeon biopsied this node as the “sentinel” and proceeded with the planned resection. Pathology later showed that the gallbladder had high-grade dysplasia (no invasive cancer), and both the cystic node and others were negative. The authors concluded that ICG-guided SLN biopsy is feasible for gallbladder neoplasms and could allow for “treatment de-escalation” in the future for select patients. They emphasize the need for further evaluation in clinical trials [27].

Overall, the evidence suggests that the sentinel node concept in GBC is not universally reliable, but it may hold true in specific scenarios (like T1 or T2 tumors not involving the liver). A completely negative cystic node in a small, early tumor might indicate the absence of spread, but surgeons must be cautious. Given the high stakes of missing metastatic nodes (which could lead to understaging and undertreatment), most guidelines still advise formal lymphadenectomy for all but the most limited cancers. For instance, even in T1b disease (which has perhaps a ~15% chance of LN metastasis), many experts recommend an extended cholecystectomy because one cannot be certain of nodal status without dissection [10].

The sentinel node studies, like Yasukawa’s, are important because they challenge the one-size-fits-all approach. They raise the possibility that in very specific subgroups of patients, we might safely do less. However, before abandoning lymphadenectomy in any patient based on a negative CLN biopsy, larger validations are needed. It is worth noting that even Yasukawa’s data, which is among the strongest for SLN in GBC, comes from a single-center retrospective study with a relatively small sample of T2p cases (only 18 patients had T2 peritoneal-side tumors). Thus, while promising, these findings should be confirmed prospectively [16].

In summary, the SLN concept in GBC is evolving. The CLN is central to this concept and has shown predictive value, especially in certain tumor locations. A positive CLN strongly implies further nodal disease (meriting full lymphadenectomy), whereas a negative CLN in a favorable context might suggest node negativity. But due to the risk of skip metastases, routine reliance on SLN biopsy alone is not yet standard. Ongoing research, including advanced imaging techniques (ICG fluorescence, perhaps PET lymphography, etc.), may further illuminate how to integrate sentinel node evaluation into GBC surgery.

Surgical and safety considerations for routine CLN excision

If one were to implement a strategy of assessing the cystic node in all gallbladder surgeries (either for diagnosing an unsuspected GBC or as part of staging), several practical questions arise: How easy is it to remove the cystic node? Does doing so increase surgical risk? What about false negatives or sampling errors? Here we address these considerations.

Identification and Excision of the Cystic Node

As discussed, the cystic node can be identified in the majority of cholecystectomies if looked for. It usually appears as a small (5-10 mm) lymph node, sometimes pigmented, in the adipose tissue of Calot’s triangle near the cystic duct. In a standard laparoscopic cholecystectomy, surgeons might or might not intentionally remove it. A study by Wysocki et al. (2018) evaluated 2,332 cholecystectomies performed by 27 surgeons and showed marked inter-surgeon variability in CLN retrieval, with rates ranging from 0% to 50% and a mean yield of 18.7%, confirming that CLN excision is highly inconsistent across surgeons and not a standardized practice. In that study, CLN retrieval was significantly less common in higher-risk patients (American Society of Anesthesiologists (ASA) ≥3) - likely reflecting surgeon caution in frailer individuals - and cases involving trainee surgeons also demonstrated lower node retrieval rates, suggesting that more conservative dissections are performed during teaching cases. Overall, the available evidence consistently shows that CLN excision during cholecystectomy is variable, generally unintentional, and influenced by surgeon experience, patient complexity, and operative context [18].

From a technical standpoint, removing the cystic node is feasible both in open and laparoscopic surgery. In an open operation, the surgeon can directly visualize and dissect it out. In laparoscopy, it requires careful dissection of Calot’s triangle beyond the critical view of safety. Surgeons must balance the desire to retrieve the node with the priority of avoiding injury to the bile ducts. Safety concerns were that the cystic node sits close to the cystic duct and artery. Aggressive dissection medially could risk clipping or cutting the common bile duct erroneously or causing hemorrhage from the portal vein or hepatic artery. However, if done carefully, the node can be excised without extra morbidity. In fact, as noted by Channa et al. (2016), keeping the dissection lateral to the cystic node is a recommended technique to avoid bile duct injury. Thus, one could mobilize the node and divide tissue around it without going medial to it. Many surgeons will actually divide the cystic artery and cystic duct, then scoop out the packet of tissue containing the cystic node from the angle between them, submitting it as a separate specimen for pathology [19].

Frozen Section and Intraoperative Assessment

In principle, if a surgeon strongly suspects GBC during an operation (for example, seeing a suspicious mass in the gallbladder), they could send the gallbladder for frozen section or the cystic node for frozen section. A positive frozen-section of the cystic node could immediately upstage the disease and might prompt a more aggressive surgery in the same sitting (such as proceeding to an open extended resection). However, incidental GBC by definition is usually not suspected at the time, so frozen sections are not routinely done. That said, if one were to adopt a routine of removing the CLN for all cholecystectomies (or all high-risk cases), sending it for pathological analysis (even if postoperatively) could provide valuable staging information early. For instance, if an unexpected GBC is found and the cystic node in the original specimen is positive, we know right away the patient is at least N1 - this might influence the urgency and extent of re-resection planning [28,29].

Does routine CLN excision cause harm? The limited data available suggest minimal downsides if done by experienced surgeons. There is no evidence that removing the cystic node prophylactically increases bile duct injuries when proper technique is followed. On the contrary, identifying the node can serve as a guide - once the node is seen, the surgeon knows the CBD lies just medial to it [30,31]. A study from Pakistan advocated that the cystic node can be a “cautionary marker”: out of 217 laparoscopic cholecystectomies, they identified the CLN in 78% and in almost all cases it lay lateral to the biliary tree; by staying lateral to the node, they could avoid bile duct injury. In their series, they were able to keep the dissection safely lateral to the node in about 46% of cases (in others, inflammation may necessitate going closer). They did not report any specific complications from dissecting around the node [19].

Another consideration is whether taking the node might compromise oncologic outcomes if cancer is present. In theory, manipulating a cancerous node could risk disseminating tumor cells, but this is a very minor concern if proper handling is done (and it would be removed en bloc). More importantly, some surgeons worry that if you take the cystic node and send it for permanent pathology, and it comes back positive, you have already “picked the cherry” - meaning the node is gone, and subsequent surgeons might want to see it in situ to guide dissection. However, this is not a major issue; if it’s positive, the patient will undergo an extended lymphadenectomy, which will clear remaining nodes anyway [12].

Use in Older or Frail Patients

One potential benefit of routine or intraoperative CLN evaluation would be in guiding management for patients who may not tolerate a full reoperation. For example, consider an 85-year-old who is found to have a T2 incidental GBC. The standard recommendation is re-resection, but if that patient has a negative cystic node and perhaps other favorable features, a multidisciplinary team might decide to forgo the risk of a major surgery and manage expectantly or with adjuvant therapy alone. Conversely, if the cystic node is positive, one might argue that the disease is systemic enough that surgery might not help (if the patient is frail). These are individualized decisions, but having the information from the cystic node could be a piece of the puzzle. As noted earlier, in practice, many elderly patients do not undergo reoperation at all - in one study, only one out of five octogenarian patients accepted re-resection when offered. Therefore, a minimally invasive sentinel node biopsy approach (for instance, via laparoscopy or even potentially endoscopic routes in the future) could be of great utility in staging such patients without subjecting them to a full laparotomy [3,9]. The 2024 case by Brañes et al. is illustrative: they had an 81-year-old woman with a suspicious gallbladder lesion and used an ICG-guided SLN biopsy to sample the node, which turned out negative. They still completed an extended cholecystectomy (as it was planned), but one could imagine in a different scenario using that information to decide on a less extensive resection if the risk-benefit calculus warranted [27].

In terms of morbidity, lymphadenectomy in the hepatoduodenal ligament can be associated with complications such as lymphatic leak, biliary injury, or hemorrhage. However, removing just the cystic node is a much more limited dissection than a full clearance of the porta hepatis. It should add only minimal operative time (a few minutes) for an experienced surgeon. If the dissection in Calot’s triangle is already being done, picking up the node is trivial in many cases. For laparoscopic surgeons not accustomed to sampling nodes, a learning curve might exist, but it’s surmountable [18,32].

Intraoperative Fluorescence or Other Technologies

The emerging use of ICG fluorescence mentioned above is a promising adjunct for identifying lymphatic drainage in real-time. It has been used in other cancers (e.g., to identify SLNs in gastric or colon surgery) [33,34]. For the gallbladder, injecting ICG into either the gallbladder wall or subserosal layer after the gallbladder is mobilized (or into the cystic artery stump) might highlight the lymphatic channel to the cystic node. This could guide the surgeon straight to the node. The 2024 report demonstrated that under near-infrared imaging, the lymphatics lit up “toward the cystic node,” and the node itself could be seen and resected laparoscopically. Importantly, that patient had no adverse effects from the ICG or the biopsy, suggesting the technique is safe and feasible even in a minimally invasive approach. As fluorescence technology becomes more widely available in ORs, this could become a tool for sentinel node mapping in iGBC [27].

In conclusion, from a surgical standpoint, routine excision of the CLN is achievable with low risk in most cholecystectomy cases. It can provide valuable staging information. The main caveat is ensuring the surgeon does not compromise the critical view of safety or injure vital structures in an attempt to retrieve the node. In difficult cases (severe inflammation or distortion of anatomy), it may be wiser to defer node dissection to an open reoperation by a hepatobiliary specialist rather than risk a bile duct injury during the index operation.

Clinical and research implications

The evolving knowledge about CLNs and sentinel node mapping in GBC carries several implications for clinical practice and future research:

Personalized Surgical Strategies

Although the historical default in iGBC has been a uniform completion operation for all tumors beyond the mucosa (≥T1b), emerging data and surgical experience support a more discriminating approach that integrates tumor side, T category, nodal biology, margin status, operative risk, and patient priorities [10]. The extremes are straightforward: T1a, margin-negative disease is cured by simple cholecystectomy, while T1b, node-negative tumors generally warrant completion extended cholecystectomy to secure accurate staging and local control, with de-escalation reserved for frail patients after multidisciplinary discussion [3]. The most credible niche for selective reduction appears to be T2 peritoneal-side disease with a proven negative cystic/sentinel node, where a parenchymal-only completion or focused lymphadenectomy may be reasonable in carefully chosen elderly or high-risk patients. In contrast, hepatic-side tumors (T2h) exhibit greater propensities for skip metastasis and microscopic liver bed invasion; here, formal portal lymphadenectomy should be maintained irrespective of cystic node negativity, and staging laparoscopy is prudent to exclude occult peritoneal disease before committing to major resection [24].

CLN positivity, at any T stage, should be interpreted as node-positive biology. In fit patients, this finding supports comprehensive nodal clearance alongside definitive local therapy; in older or comorbid individuals, it justifies prioritizing systemic therapy first with response-adapted consideration of surgery. Radiologically suspicious basin nodes with nondiagnostic tissue sampling should not be “rescued” by a negative or indeterminate sentinel node, given the false-negative risk; most teams proceed to completion resection with formal lymphadenectomy when technically feasible [10,12]. When high-morbidity resections are anticipated (e.g., major hepatectomy and/or bile duct resection) or the anatomy is borderline (T3/T4, bulky hepatic-side nodal disease), a short neoadjuvant interval - typically a gemcitabine-platinum doublet - can test tumor biology, treat micrometastases early, and spare non-beneficial surgery in early progressors; restaging with high-quality CT/MRI and, where appropriate, laparoscopy should precede any attempt at R0 resection. Margin-positive (R1) status after completion of resection warrants consideration of re-resection when feasible; if not, a planned course of adjuvant chemoradiation following systemic therapy addresses the heightened risk of locoregional failure. Finally, intraoperative suspicion at the index cholecystectomy should prompt targeted frozen sections and submission of the CLN - without compromising the critical view of safety - and bile spillage or perforation should tilt subsequent management toward thorough lymphadenectomy, adjuvant chemotherapy for ≥T2 disease, and intensified surveillance. This kind of risk stratification is a major goal of ongoing research [11,35].

Intraoperative Decision-Making

The concept of checking the cystic node during the initial cholecystectomy for suspicion of cancer could become more common. While truly incidental cancers are unexpected, there are sometimes intraoperative clues (e.g., a suspicious hard mass in the gallbladder, or porcelain gallbladder) where a surgeon might suspect cancer. In those cases, doing a frozen section of the gallbladder or cystic node can be considered. A positive finding could prompt an immediate extension of surgery under the same anesthesia - for example, conversion from laparoscopic to open and performing an extended resection then and there. This avoids a second surgery and potential tumor seeding during the interval. However, this approach requires that the hospital have pathology support on standby and a surgeon skilled in hepatobiliary surgery present or available. In practice, immediate re-resection is not commonly done for iGBC unless it was strongly suspected preoperatively [3]. Nonetheless, as techniques like ICG SLN mapping improve, one could envision a scenario where, during a routine cholecystectomy, the surgeon injects ICG, identifies the sentinel node, excises it, and does a frozen section [27]. If it’s positive, they proceed to an extended resection in the same operation. If negative and the tumor is small (T1b/T2) on frozen, perhaps they will conclude the operation, knowing the likelihood of further spread is low. Such an approach remains investigational but is worth exploring [3].

Guideline Refinement

At present, major guidelines (e.g., the National Comprehensive Cancer Network (NCCN), the European Society for Medical Oncology (ESMO), Japanese Biliary Society) emphasize formal reoperation for iGBC T1b+ with lymphadenectomy. The data from Yasukawa et al. and others might eventually inform guideline updates to nuance that recommendation. For example, the Japanese guidelines might incorporate tumor location into their advice (there is already some suggestion of that in practice in high-volume centers) [10,13,36]. If future studies corroborate that peritoneal-side T2 cancers rarely have metastases beyond a negative CLN, guidelines might allow a less radical approach for those specific cases. Any such change would require strong evidence, likely from prospective trials or at least large observational studies.

Research Directions

There is a clear need for prospective research on SLN mapping in GBC. A possible trial design could be: enroll patients with suspected early-stage GBC (maybe incidentally found on imaging or at surgery), perform SLN biopsy with a technique like ICG or dye, and compare the pathology of SLN to the completion lymphadenectomy. Endpoints would be the sensitivity and negative predictive value of the SLN. If the SLN biopsy can accurately predict nodal status, then a second phase could even test a strategy of SLN biopsy alone versus full lymphadenectomy in certain patients. Given the rarity of GBC, such studies would have to be multi-institutional and likely international.

Another area of research is molecular analysis of the cystic node. For example, ultrastaging with immunohistochemistry or molecular assays (as used in melanoma or breast SLNs) could potentially detect micrometastases that routine H&E might miss. This could further refine staging - although if micrometastases are found, the patient would likely be treated as positive anyway.

Oncologic Outcomes and Adjuvant Therapy

Precision in nodal staging-augmented by CLN/sentinel node assessment, where informative, aligns patients to appropriately scaled perioperative therapy. As a rule, resected ≥T2 tumors or any node-positive disease merit adjuvant chemotherapy, with a fluoropyrimidine backbone commonly administered for six months; margin-positive resections prompt consideration of consolidative chemoradiation after systemic therapy or, when feasible, re-resection to R0. By contrast, most T1b N0 R0 cases require no adjuvant therapy, and T1a R0 never does. A positive sentinel/CLN upstages the patient to N+ and therefore mandates adjuvant treatment even if other nodes are negative, whereas a negative CLN - though reassuring - does not negate chemotherapy for ≥T2 disease. Neoadjuvant chemotherapy is not routine for clearly resectable iGBC but is reasonable in T3/T4 presentations, in hepatic-side disease with bulky nodes, or when major resection is anticipated; patients are typically restaged after two to four cycles, proceeding to surgery only if disease is controlled and performance status remains favorable [10,37]. This is another facet where precision in nodal staging matters.

Cost-Benefit Considerations

A shift to routine CLN excision or SLN mapping should also be justified by improved outcomes or cost-effectiveness. One could argue that adding an ICG kit and 20 minutes of mapping time to a cholecystectomy might save a major surgery later. If, for instance, 100 cholecystectomy patients need mapping to correctly identify five who can avoid a big reoperation, is that worthwhile? Given the morbidity and costs of an extended hepatectomy, it possibly is [16,33]. However, these calculations will require data on how many patients can be spared surgery or have improved survival by such strategies.

**Figure 1:**
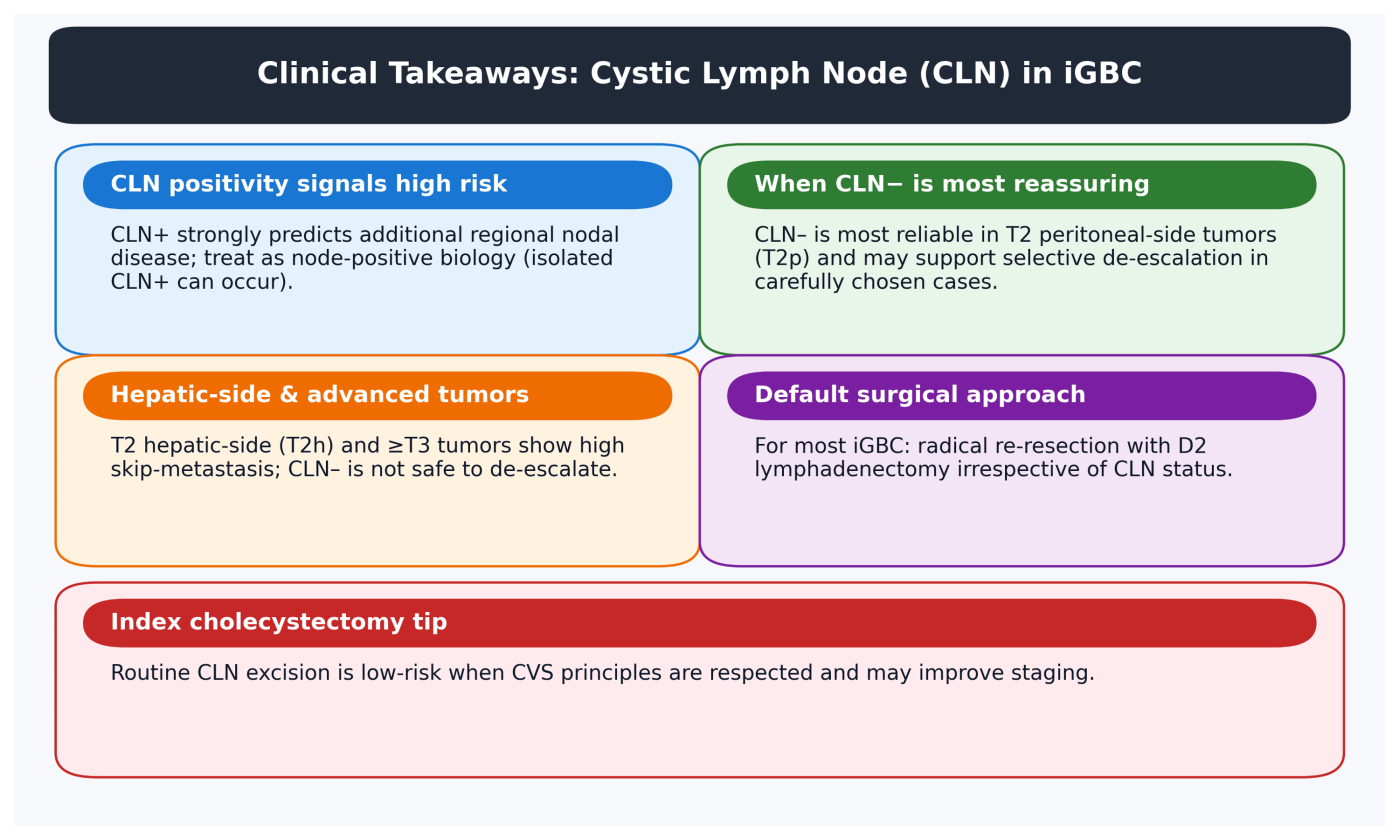
Clinical takeaways for cystic lymph node (CLN) assessment in incidental gallbladder cancer (iGBC). CVS: critical view of safety Credit: The image was created by the authors.

In summary, the clinical implications of recognizing the cystic node’s role are significant. Right now, every incidental GBC beyond T1a is treated aggressively because we lack a reliable way to tell who truly needs it. The sentinel node concept, if validated, could become a game-changer in tailoring therapy - doing more for those who need it and less for those who don’t. Until then, the prudent approach is to continue standard management (complete resection with nodes) for fit patients, but to incorporate cystic node analysis in our evaluations to gather more data. For practical application, the key takeaways regarding CLN reliability and surgical implications are summarized in Figure 1.

## Conclusions

iGBC presents a unique clinical scenario where surgical management must balance oncologic thoroughness with patient safety. The CLN, as the first station in gallbladder lymphatic drainage, plays a pivotal role in the spread and staging of the disease. Evidence to date confirms that involvement of the cystic node often heralds more extensive nodal metastasis and portends worse outcomes, whereas a negative cystic node is an encouraging sign associated with a lower likelihood of widespread nodal disease. However, the SLN concept in GBC is only partially reliable - skip metastases can occur, especially in more advanced tumors or those with unfavorable anatomy (e.g., touching the liver). Therefore, a negative CLN cannot uniformly guarantee that a patient is node-negative, and the current standard of care remains an extended resection with comprehensive lymphadenectomy for most patients with iGBC. That said, emerging research offers a glimpse of a more nuanced approach. In carefully selected cases - notably T2 tumors confined to the peritoneal side of the gallbladder - a negative cystic node may truly indicate absence of further metastasis, raising the potential to modify the surgical extent in such situations. Advanced techniques like ICG-guided SLN biopsy demonstrate that it is technically feasible to identify and sample the cystic node minimally invasively. These innovations pave the way for future protocols where the decision to perform a radical resection could be informed by intraoperative nodal assessment.

For now, surgeons managing incidental GBC should ensure a meticulous evaluation of the cystic node - either by having the pathologist identify it in the cholecystectomy specimen or by excising it if visibly enlarged or suspicious. Every piece of information matters for staging and planning further treatment. Routine excision of the CLN during cholecystectomy is a low-risk addition that can aid pathology in identifying nodal involvement early. In the event of a positive cystic node, there is unanimous agreement that a formal lymph node dissection and appropriate adjuvant therapy are indicated. In the event of a negative cystic node (and especially if the tumor is early-stage), the treating team should still adhere to guidelines but can counsel patients that the prognosis is correspondingly better. Ultimately, the goal is to improve patient outcomes by tailoring interventions to disease extent. The CLN - once considered only an anatomic curiosity in Calot’s triangle - has emerged as a key sentinel guiding the surgeon’s hand. Its routine excision and examination of iGBC should be viewed as a useful practice that complements systemic staging. While it is not yet time to abandon proven surgical principles, incorporating the growing evidence on CLN and SLN concepts will refine our approach. Ongoing research and possibly prospective trials will determine whether select patients with negative sentinel nodes can be safely spared an extensive reoperation. Until then, the management of iGBC should remain vigilant: thorough surgical resection (including CLN excision) and diligent pathological assessment are paramount. By doing so, we honor both the tenets of oncologic surgery and the promise of precision medicine, ensuring each patient receives the right level of intervention for their disease.
